# Aerosol exposure of staff during dental treatments: a model study

**DOI:** 10.1186/s12903-022-02155-9

**Published:** 2022-04-15

**Authors:** Florentina Melzow, Sarah Mertens, Hristo Todorov, David A. Groneberg, Sebastian Paris, Alexander Gerber

**Affiliations:** 1 Department of Operative, Preventive and Pediatric Dentistry, Center of Oral Health Sciences, Charité – Universitätsmedizin Berlin, Aßmannshauser Str. 4-6, 14197 Berlin, Germany; 2 Institute of Human Genetics, University Medical Center Mainz, Mainz, Germany; 3 Institute of Occupational, Social and Environmental Medicine, Goethe University Frankfurt, Frankfurt am Main, Germany

**Keywords:** Aerosols, Oral medicine, Dental staff, Dental suction, COVID-19, SARS-CoV-2

## Abstract

**Background:**

Due to exposure to potentially infectious aerosols during treatments, the dental personnel is considered being at high risk for aerosol transmitted diseases like COVID-19. The aim of this study was to evaluate aerosol exposure during different dental treatments as well as the efficacy of dental suction to reduce aerosol spreading.

**Methods:**

Dental powder-jet (PJ; Air-Flow^®^), a water-cooled dental handpiece with a diamond bur (HP) and water-cooled ultrasonic scaling (US) were used in a simulation head, mounted on a dental unit in various treatment settings. The influence of the use of a small saliva ejector (SE) and high-volume suction (HVS) was evaluated. As a proxy of aerosols, air-born particles (PM10) were detected using a Laser Spectrometer in 30 cm distance from the mouth. As control, background particle counts (BC) were measured before and after experiments.

**Results:**

With only SE, integrated aerosol levels [median (Q25/Q75) µg/m^3^ s] for PJ [91,246 (58,213/118,386) µg/m^3^ s, *p* < 0.001, ANOVA] were significantly increased compared to BC [7243 (6501/8407) µg/m^3^ s], whilst HP [11,119 (7190/17,234) µg/m^3^ s, *p* > 0.05] and US [6558 (6002/7066) µg/m^3^ s; *p* > 0.05] did not increase aerosol levels significantly. The use of HVS significantly decreased aerosol exposure for PJ [37,170 (29,634/51,719) µg/m^3^ s; *p* < 0.01] and HP [5476 (5066/5638) µg/m^3^ s; *p* < 0.001] compared to SE only, even reaching lower particle counts than BC levels for HP usage (*p* < 0.001).

**Conclusions:**

To reduce the exposure to potentially infectious aerosols, HVS should be used during aerosol-forming dental treatments.

## Background

The WHO classified the global spreading of SARS-CoV-2 as a pandemic in March 2020 [1]. This is the third major outbreak of a Corona virus after the Severe Acute Respiratory Syndrome (SARS) pandemic in 2003 and the Middle East Respiratory Syndrome (MERS) in 2012 [2, 3]. The course of COVID-19 varies greatly. While many cases in younger individuals are mild or even symptom-free, more severe cases are observed in older patients and about 5% of patients develop progressive respiratory failure requiring intensive medical treatment [4]. The rapid spread of COVID-19 causes a considerable burden on the health systems of the affected countries. Therefore, many governments worldwide have repeatedly ordered social lockdowns, to slow down the rapid spread of COVID-19 and avoid medical systems to collapse.

COVID-19 as many other respiratory infections is considered to be primarily transmitted airborne via droplets and aerosols [5]. Aerosols are defined as a dispersion of particles dissolved in gas. They are found in almost every environment and can be natural, such as pollen, or man-made, such as particulate matter from combustion processes. Due to their small size, they remain airborne for a long time [6]. Droplets are larger than aerosols and tend to settle faster [7]. The size of aerosols is not conclusively defined and, depending on the publication, usually varies from less than 5 µm to less than 50 µm in diameter [8]. While larger droplets carry a greater virus load and tend to deposit in the upper airway, smaller aerosols penetrate deep into the lungs [9]. Depending on room conditions, larger droplets may evaporate into smaller droplet nuclei that remain in the environment for several hours and can be moved by air draught [10]. Many routine dental procedures, such as the use of rotary dental instruments, ultrasonic scalers or air–water syringes, produce visible sprays and thus probably large amounts of aerosols. Due to the close proximity to the patient, dental staff are likely to be exposed to both rapidly settling droplets and aerosols [11], and thus at increased risk of COVID-19 infection. Moreover, the salivary glands and saliva itself have been identified as important virus reservoirs, which makes the saliva-contaminated dental aerosol appear to be a potential source of infection [12–14]. Since the beginning of the pandemic, many studies have been conducted on the generation and elimination of dental aerosols [7, 15, 16]. Due to the heterogeneity of the methodologies used, the evidence base is still limited and requires further research. Many of the studies to date show contamination of the environment via microbiological cultivation or dye detection, but there have been only few investigations into the quantity and quality of the aerosol produced. The influence on the exposure due to the positioning of the patient and the elimination of the aerosol have also hardly been investigated so far [10].

Due to this uncertainty of the potential hazard of dental aerosols as infection for dental personnel as well as patients, many countries ordered the temporary closure of dental surgeries during the lockdown to avoid super spreading events. While many dental procedures can be postponed for a while, there are also dental emergencies that require immediate treatment. This and cases of undetected infections lead to an unclear risk situation for dental staff.

In the context of this current pandemic with relation to dentistry, the question arises, as to what extent dental personnel are at risk at their working environment. Due to the work with potentially infectious body fluids and the physical proximity between dentist and patient, gloves, surgical masks and protective goggles are standard hygiene measures. These provide the practitioner with considerable protection from potentially infectious droplets. Since the beginning of the pandemic, dental protective equipment has been extended in many countries, including FFP2/3-masks (N95), face shields and surgical bonnets [17]. But even FFP2-masks cannot guarantee complete protection from aerosols [18, 19].

Reports from different countries indicate, that the currently used personnel protective equipment seems to be effectively protecting the dental personnel [20]. Also, the prevalence for COVID-19 in American dentists remained under the rate for the general population [21]. Do aerosols after all play a less important role in the transmission of SARS-CoV-2 or is the exposure of dental staff less than expected?

The aim of this study was to evaluate aerosol exposure in different dental settings, varying in treatment method, dental suction and patient position. Our null-hypotheses were, that there are no significant differences in aerosol exposure between different (a) treatment methods, (b) suction methods and (c) patient positions.

## Methods

As a proxy for aerosol exposure in dental practice, airborne particles of 10 µm diameter and less (PM10 including the smaller fractions of PM2.5 and PM1) were detected using a Laser Aerosol Spectrometer (MINI-LAS 11-R, Grimm Technologies, Ainring, Germany) with ISO 21501-1 in a standardized patient model. The influence of the treatment method (powder jet tooth cleaning, high-speed dental handpiece preparation, ultrasonic scaling), suction (no suction, small saliva ejector, high-volume suction) and patient position (upper, lower jaw, anterior, posterior) on aerosol levels was evaluated.

### Experimental set-up

Measurements were performed in a standard treatment 22 m^2^ room at the Center for Oral Health Sciences at Charité Berlin, on ten different days between June and October 2020. Ambient conditions were largely constant on these days (24 ± 4 °C room temperature, 58–75% humidity and 0 m/s air movement, closed windows, no air conditioning). To simulate real-life conditions, no further attempts were taken to standardise ambient conditions.

Two experienced and calibrated right-handed dentists alternately performed all simulated different dental settings on a phantom head (P-6/5 TSE, frasaco, Tettnang, Germany) with training teeth (Standard Series AG-3, frasaco) mounted on a dental unit (Teneo, Dentsply Sirona, Bensheim, Germany). We simulated three dental treatment modalities: first, powder jet tooth cleaning (PJ; Airflow Prophylaxis Master, level 4, E.M.S., Nyon, Switzerland) with Prophylaxis Powder (Airflow Plus; E.M.S.) was performed. Second, high-speed dental handpiece preparation (HP; T1 Line, Dentsply Sirona) was performed with a fine, cylindrical dental bur (8879L, Komet Dental, Lemgo, Germany) at 40,000 rpm. Third, ultrasonic scaling (US; SiroSonic TL^2^, Dentsply Sirona) was performed at maximum vibration strength with Tip no. 1L (Dentsply Sirona). Water supply for each device was set on maximum (PJ: 90 ml/min; HP: 40 ml/min US: 60 ml/min).

To simulate varying angles and positions in all settings, for each experimental set-up, treatment was performed on eight different teeth (FDI code: 16, 12, 22, 26, 36, 32, 42, 46). Dentist´s treatment position varied between 12 o’clock (maxilla) and 9–10 o’clock (mandible). If used, the saliva ejector (SE; approx. 3.3 l/min) (HS-Speichelsauger, Henry Schein, Langen, Germany) was placed angled in the opposite corner of the mouth. The high-volume suction (HVS; approx. 360 l/min) (Universalkanüle, Dürr Dental SE, Bietigheim-Bissingen, Germany) was positioned directly at the treated tooth and always used in combination with the small SE. The dentists held the HVS device themselves without assistance. A summary of the experimental set-up is shown in Table 1.

**Table 1:** Experimental set-up

Setting	Treatment modality	Suction	n
1	Dental powder-jet	No suction	8
2		Saliva ejector	8
3		High-volume-suction	8
4	High-speed handpiece	No suction	8
5		Saliva ejector	8
6		High-volume-suction	8
7	Ultrasonic scaling	No suction	8
8		Saliva ejector	8
9		High-volume-suction	8

**Fig. 1:**
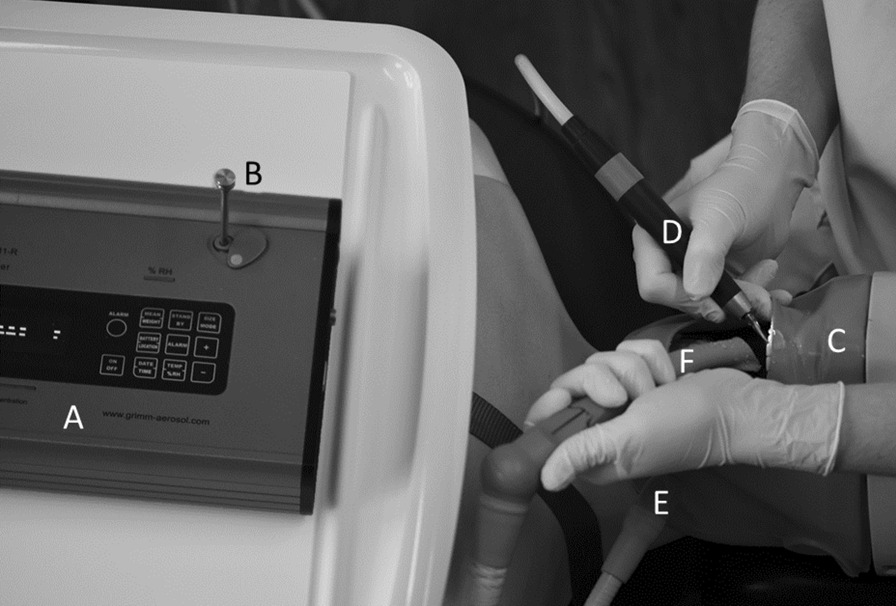
Scheme of experimental set-up. **A** Laser spectrometer, **B** inlet laser spectrometer, **C** phantom head, **D** high-speed handpiece, **E** saliva ejector, **F** high volume suction

Concentration of airborne particles (PM10) was recorded, using a Laser Aerosol Spectrometer with a time resolution of 6 s. The spectrometer was positioned in front of the patient at a distance of 30 cm from the oral cavity, representing the minimum distance to simulate maximum exposure of dental personnel (Fig. 1). The distance of the spectrometer was controlled and corrected when the patient was repositioned for treatment of the maxilla or mandible. Background aerosol levels were recorded over a period of 5 min repeatedly before and after each exposure measurement. To reset background levels between experimental set-ups, windows were opened, until background levels were reached. Meanwhile the spectrometer run an automatic cleaning program. For each of the tested settings (Table 1) eight measurements were performed at the above-mentioned teeth. Each measurement lasted 5 min with continuous treatment, separated by a break of 1 min, respectively. After completion of one setting the room was ventilated again, followed by another background measurement. Temperature, humidity and air movement data were continuously recorded.

### Statistical analysis

Acquired data were transferred to a computer and recorded (Microsoft Excel 2019, Microsoft, Redmond, WA, USA). The area under the curve of PM10 (AUC_PM10_ in µg/m^3^s) as a plot of PM10 in breathing air against time was calculated exemplarily for a period of 5 min [22].

Statistical analysis was performed with R 4.0.2 (R Foundation for Statistical Computing, Vienna, Austria). Results were represented graphically as Log_e_ Area under Curve PM10 Box-and-whisker-plots. More than two groups were compared statistically using two-way ANOVA followed by Tukey comparisons of marginal means. Welch’s ANOVA followed by Tamhane–Dunnett Many-to-One Comparison Test (due to unequal variances in different groups) to compare background values versus treatment modalities. The original values were transformed using the natural logarithm in case that the data violated the assumptions of normality of the residuals and variance homogeneity. *p* values were two-tailed and the threshold for statistical significance was set to a = 0.05.

## Results

**Fig. 2:**
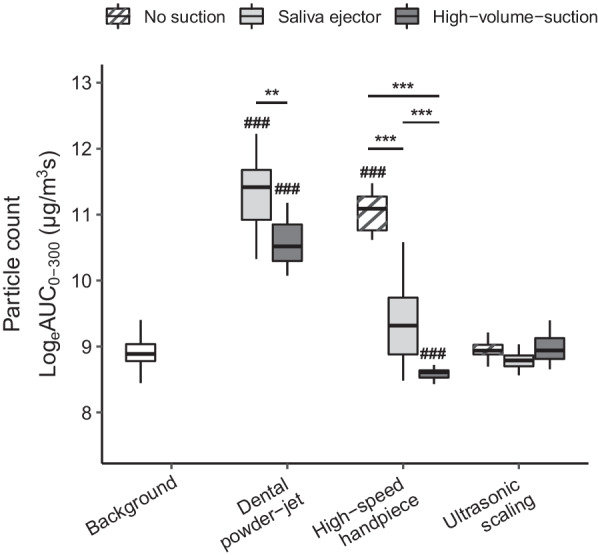
Results of measured aerosol concentrations with different suction types, grouped by procedure. Results shown as Log_e_ Area under Curve PM10 Box-and-whisker-plots (box and thick line = 25th/75th percentiles and median, error bars = minima and maxima). Significant differences with background are indicated with hashes: # *p* < 0.05, ### *p* < 0.001; 1-way Welch’s ANOVA followed by Tamhane–Dunnett Many-to-One Comparison Test. Significant differences within treatment modalities are indicated with asterisks: **p < 0.01, ***p < 0.001; two-way ANOVA followed by Tukey comparisons of marginal means

A mean AUC (SD) background level 7243 [median (Q25/Q75) (6501/8407)] µg/m^3^s was determined from the recorded background aerosol levels before, during and after experiments. During dental treatments particle counts in 30 cm distance were increased up to 32-fold of background level (Fig. 2). When comparing the three treatments, powder jet tooth cleaning showed highest particle counts, followed by handpiece usage and ultrasonic scaling. Powder-jet could not be used without any suction, as this filled the room with particles immediately. For ultrasonic scaling particle counts did not exceed background levels in all settings (with or without suction) (Fig. 2).

The use of the small saliva ejector reduced particle counts significantly for handpiece usage, while the saliva ejector and high-volume suction in combination significantly reduced values for powder-jet and handpiece (*p* < 0.001, ANOVA), even reaching particle counts significantly lower than background levels for the latter (*p* < 0.001, ANOVA) (Fig. 2).

## Discussion

Infectious diseases affecting the respiratory tract, like COVID-19 are considered to be spread via direct contact, droplets and aerosols [23]. As dental treatments necessarily involve contact with potentially contagious liquids and droplet as well as aerosol exposure, the dental personal but also subsequent patients might be at higher risk of acquiring nosocomial infections.

While high particle counts for high-speed dental handpiece preparation and powder-jet tooth cleaning were expected, the low aerosol exposure of ultrasonic scaling was rather surprising, as during treatment the water spray is even visible with the naked eye and significant amounts of cooling liquid can be detected in the field around the patient after treatment [24]. Therefore, prophylaxis and periodontal therapy were considered being associated with a higher risk of COVID-19 transmission compared with other treatments [25]. The current data put this in question. A plausible explanation for the observed low aerosol exposure could be that the ultrasonic-generated water spray consists of rather big droplets, which sediment rapidly and are not detected with our methodology as we only measured particles with 10 µm diameter or smaller. In addition, studies have shown that there is already a significant reduction in particle size at a distance of 0.5 m. This effect seems to be further enhanced by suction [26, 27]. One recently published article found only low evidence for transmission prevention due use of the HVE [16]. Some studies found beneficial effects for dental suction at already low or intermediate flow rates, which corroborates our results [28].

Our study has some limitations and several aspects require discussion. First, in order to standardize patient parameters, we chose for a simulated mannequin patient model which cannot simulate all factors affecting aerosol exposure of the personnel. For example, patient´s breath was not simulated although the exhalation might accelerate airborne particles, resulting in increased aerosol exposure levels. At the same time, we did not control all environmental settings as these were not the focus of the current study. Rather, we performed measurements in real life conditions. In different environmental settings, parameters such as room size, air movement and exchange, humidity aerosol exposure might differ.

Second, we measured PM10 particle counts as a proxy for potentially infections aerosols that are emitted during dental treatments. However, as the great majority of the emitted spay during dental treatments consists of cooling water or powder particles, the PM10-values, we measured cannot be equalized with potentially infectious aerosols that are emitted during exhalation or coughing [29, 30]. It is currently unclear, what fraction of dental aerosols consist of potentially infectious liquids like saliva or blood. It is likely however, that body fluids only account for minor fractions of the aerosols and consequently bacteria or virus concentrations in aerosol droplets are low compared to respiration generated aerosols. Reports show possible transmission of viral diseases bound to fine particles [31, 32]. This suggests that transmission through powder particles is possible, even if it can be assumed that the proportion of infectious components is low compared to a large amount of carrier substance. Therefore, our results do not allow direct determination of the health hazard arising from dental treatments. However, they are useful to compare the relative risk at different treatment settings and impact on suction to reduce aerosol exposure.

The influence of relative humidity on PM10 is partly due to the operating principle of the used spectrometer. The device works with the scattered light method and thus is susceptible to changes in relative humidity due to increase of mass and diameter of the measured particulate matter as vapor condenses on dust particles or vapor droplets connect to larger units and lead to stronger deflection of the measuring laser. It should be considered that humidity as well as temperature and of course ventilation have a significant impact on virus spreading via aerosols [5, 17, 33]. Therefore, these parameters should be more closely evaluated to estimate the risk and to seize appropriate protective measures.

We decided to measure PM10 and not additionally PM5, PM2.5 or PM1 as performed for several tobacco smoke studies [34, 35] as PM10 also includes the smaller PM fractions. Particles larger than 10 µm in aerodynamic diameter tend to sediment quickly and are less responsible for the exposure to long-lasting aerosols in the dental treatment room. Particles of the PM2.5 and PM1 fractions are also more likely to cause long-lasting aerosols but mainly penetrate into the deep airways and the lungs [36], while larger particles tend to deposit in the upper airways [37], which have proven to be the main entry point for SARS-CoV-2 [38]. While smaller particles remain longer in the air, their volume decreases strongly with a reduced diameter [19]. The infectious potential therefore is presumably lower, as the number of virus particles contained decreases with size [39].

The risk of infection from droplets and aerosols in the dental field remains unclear. While some studies show a higher risk of transmission by droplets than by aerosols [40] other also show a high risk of infections via aerosols [10]. However, droplets are easier to control than aerosols through appropriate protective clothing and hygiene protocols. The commonly used four-hand technique, in which the suction devices are used by the dental assistant, probably leads to even better aerosol reduction results. Our study supports the data situation for aerosol exposure of dental staff by measurements with the laser spectrometer, in particular under consideration of different suction methods. Measurements with the laser spectrometer can depict aerosol development more accurately than dye or microbiological methods [10]. Air movements and humidity have a significant influence on the behaviour of the resulting aerosols. Further research related to these circumstances and, in particular, the behaviour of SARS-CoV-2 are critical to make conclusions about the risk of infection for dental practitioners.

## Conclusions

Water-cooled dental treatments such as powder jet tooth cleaning and high-speed hand piece preparation produce significant amounts of aerosols. It is not clear yet to what fraction these aerosols contain potentially infectious liquids like saliva or blood. However, high-volume dental suction may significantly reduce aerosol spread and therefore should be used whenever possible to prevent aerosol-transmitted infections during dental treatments.

## Data Availability

The raw data is a property of Charité – Universitätsmedizin Berlin and Goethe University Frankfurt. The data set used and analyzed during the current study are available from corresponding author on reasonable request.

## Abbreviations

PJ: Dental powder-jet
HP: Dental handpiece
US: Ultrasonic scaling
SE: Saliva ejector
HVS: High-volume suction
PM10: Air-born particles
BC: Background particle counts

## References

[1] Ahmed MA, Jouhar R, Ahmed N, Adnan S, Aftab M, Zafar MS, Khurshid Z (2020). Fear and practice modifications among dentists to combat novel coronavirus disease (COVID-19) outbreak. Int J Environ Res Public Health.

[2] Drosten C, Gunther S, Preiser W, van der Werf S, Brodt HR, Becker S, Rabenau H, Panning M, Kolesnikova L, Fouchier RA (2003). Identification of a novel coronavirus in patients with severe acute respiratory syndrome. N Engl J Med.

[3] Zaki AM, van Boheemen S, Bestebroer TM, Osterhaus AD, Fouchier RA (2012). Isolation of a novel coronavirus from a man with pneumonia in Saudi Arabia. N Engl J Med.

[4] Wu Z, McGoogan JM (2020). Characteristics of and important lessons from the coronavirus disease 2019 (COVID-19) outbreak in China: summary of a report of 72,314 cases From the Chinese Center for Disease Control and Prevention. JAMA.

[5] Setti L, Passarini F, De Gennaro G, Barbieri P, Perrone MG, Borelli M, Palmisani J, Di Gilio A, Piscitelli P, Miani A (2020). Airborne transmission route of COVID-19: why 2 m/6 feet of inter-personal distance could not be enough. Int J Environ Res Public Health.

[6] Kumar PS, Subramanian K (2020). Demystifying the mist: sources of microbial bioload in dental aerosols. J Periodontol.

[7] Nobrega MTC, Bastos R, Mecenas P, de Toledo IP, Richardson-Lozano R, Altabtbaei K, Flores-Mir C (2021). Aerosol generated by dental procedures: a scoping review. J Evid Based Med.

[8] Johnson IG, Jones RJ, Gallagher JE, Wade WG, Al-Yaseen W, Robertson M, McGregor S, Sukriti KC, Innes N, Harris R (2021). Dental periodontal procedures: a systematic review of contamination (splatter, droplets and aerosol) in relation to COVID-19. BDJ Open.

[9] Kohanski MA, Palmer JN, Cohen NA (2020). Aerosol or droplet: critical definitions in the COVID-19 era. Int Forum Allergy Rhinol.

[10] Innes N, Johnson IG, Al-Yaseen W, Harris R, Jones R, Kc S, McGregor S, Robertson M, Wade WG, Gallagher JE (2021). A systematic review of droplet and aerosol generation in dentistry. J Dent.

[11] Ge ZY, Yang LM, Xia JJ, Fu XH, Zhang YZ (2020). Possible aerosol transmission of COVID-19 and special precautions in dentistry. J Zhejiang Univ Sci B.

[12] Azzi L, Carcano G, Gianfagna F, Grossi P, Gasperina DD, Genoni A, Fasano M, Sessa F, Tettamanti L, Carinci F (2020). Saliva is a reliable tool to detect SARS-CoV-2. J Infect.

[13] Baghizadeh Fini M (2020). Oral saliva and COVID-19. Oral Oncol.

[14] Alqutaibi AY, Saeed MH, Aboalrejal AN (2021). Saliva may be considered as reliable tool for diagnosis of COVID-19 when compared with nasopharynx or throat swabs. J Evid Based Dent Pract.

[15] Kun-Szabo F, Gheorghita D, Ajtai T, Hodovany S, Bozoki Z, Braunitzer G, Antal MA (2021). Aerosol generation and control in the dental operatory: an in vitro spectrometric study of typical clinical setups. PLoS ONE.

[16] Kumbargere Nagraj S, Eachempati P, Paisi M, Nasser M, Sivaramakrishnan G, Verbeek JH (2020). Interventions to reduce contaminated aerosols produced during dental procedures for preventing infectious diseases. Cochrane Database Syst Rev.

[17] Villani FA, Aiuto R, Paglia L, Re D (2020). COVID-19 and dentistry: prevention in dental practice, a literature review. Int J Environ Res Public Health.

[18] Sommerstein R, Fux CA, Vuichard-Gysin D, Abbas M, Marschall J, Balmelli C, Troillet N, Harbarth S, Schlegel M, Widmer A (2020). Risk of SARS-CoV-2 transmission by aerosols, the rational use of masks, and protection of healthcare workers from COVID-19. Antimicrob Resist Infect Control.

[19] Deutsche Gesellschaft für Zahn- M-Ukd. Umgang mit zahnmedizinischen Patienten bei Belastung mit Aerosol-übertragbaren Erregern. In*:* vol. 083-046: AWMF online; 2020:44.

[20] Nardone M, Cordone A, Petti S (2020). Occupational COVID-19 risk to dental staff working in a public dental unit in the outbreak epicenter. Oral Dis.

[21] Araujo MWB, Estrich CG, Mikkelsen M, Morrissey R, Harrison B, Geisinger ML, Ioannidou E, Vujicic M (2021). COVID-2019 among dentists in the United States: a 6-month longitudinal report of accumulative prevalence and incidence. J Am Dent Assoc.

[22] Gerber A, Bigelow A, Schulze M, Groneberg DA (2015). Brand cigarillos—a cheap and less harmful alternative to cigarettes? Particulate matter emissions suggest otherwise. Int J Environ Res Public Health.

[23] Jones RM, Brosseau LM (2015). Aerosol transmission of infectious disease. J Occup Environ Med.

[24] Graetz C, Bielfeldt J, Tillner A, Plaumann A, Dorfer CE (2014). Spatter contamination in dental practices—how can it be prevented?. Rev Med Chir Soc Med Nat Iasi.

[25] Jungo S, Moreau N, Mazevet ME, Ejeil AL, Biosse Duplan M, Salmon B, Smail-Faugeron V (2021). Prevalence and risk indicators of first-wave COVID-19 among oral health-care workers: a French epidemiological survey. PLoS ONE.

[26] Allison JR, Currie CC, Edwards DC, Bowes C, Coulter J, Pickering K, Kozhevnikova E, Durham J, Nile CJ, Jakubovics N (2021). Evaluating aerosol and splatter following dental procedures: addressing new challenges for oral health care and rehabilitation. J Oral Rehabil.

[27] Yang M, Chaghtai A, Melendez M, Hasson H, Whitaker E, Badi M, Sperrazza L, Godel J, Yesilsoy C, Tellez M (2021). Mitigating saliva aerosol contamination in a dental school clinic. BMC Oral Health.

[28] Singh H, Maurya RK, Sharma P, Kapoor P, Mittal T (2021). Aerosol generating procedural risks and concomitant mitigation strategies in orthodontics amid COVID-19 pandemic—an updated evidence-based review. Int Orthod.

[29] Fabian P, Brain J, Houseman EA, Gern J, Milton DK (2011). Origin of exhaled breath particles from healthy and human rhinovirus-infected subjects. J Aerosol Med Pulm Drug Deliv.

[30] Yang S, Lee GW, Chen CM, Wu CC, Yu KP (2007). The size and concentration of droplets generated by coughing in human subjects. J Aerosol Med.

[31] Chen G, Zhang W, Li S, Zhang Y, Williams G, Huxley R, Ren H, Cao W, Guo Y (2017). The impact of ambient fine particles on influenza transmission and the modification effects of temperature in China: a multi-city study. Environ Int.

[32] Zhao Y, Richardson B, Takle E, Chai L, Schmitt D, Xin H (2019). Airborne transmission may have played a role in the spread of 2015 highly pathogenic avian influenza outbreaks in the United States. Sci Rep.

[33] Inthavong K, Ge QJ, Li XD, Tu JY (2012). Detailed predictions of particle aspiration affected by respiratory inhalation and airflow. Atmos Environ.

[34] Gerber A, Hofen-Hohloch AV, Schulze J, Groneberg DA (2015). Tobacco smoke particles and indoor air quality (ToPIQ-II)—a modified study protocol and first results. J Occup Med Toxicol.

[35] Wasel J, Boll M, Schulze M, Mueller D, Bundschuh M, Groneberg DA, Gerber A (2015). Brand cigarillos: low price but high particulate matter levels—is their favorable taxation in the European Union justified?. Int J Environ Res Public Health.

[36] Duguid JP (1946). The size and the duration of air-carriage of respiratory droplets and droplet-nuclei. J Hyg (Lond).

[37] Ge QJ, Inthavong K, Tu JY (2012). Local deposition fractions of ultrafine particles in a human nasal-sinus cavity CFD model. Inhal Toxicol.

[38] Hou YJ, Okuda K, Edwards CE, Martinez DR, Asakura T, Dinnon KH, Kato T, Lee RE, Yount BL, Mascenik TM (2020). SARS-CoV-2 reverse genetics reveals a variable infection gradient in the respiratory tract. Cell.

[39] Stadnytskyi V, Bax CE, Bax A, Anfinrud P (2020). The airborne lifetime of small speech droplets and their potential importance in SARS-CoV-2 transmission. Proc Natl Acad Sci U S A.

[40] Meng L, Hua F, Bian Z (2020). Coronavirus disease 2019 (COVID-19): emerging and future challenges for dental and oral medicine. J Dent Res.

[41] Abstracts of the 10th Virtual Conseuro 2021 Congress. Clin Oral Investig. 2021:4185–238. 10.1007/s00784-021-03940-6 PMC8060788 33885996

